# An Evolutionary Mechanism for the Generation of Competing RNA Structures Associated with Mutually Exclusive Exons

**DOI:** 10.3390/genes9070356

**Published:** 2018-07-17

**Authors:** Timofei M. Ivanov, Dmitri D. Pervouchine

**Affiliations:** 1 Skolkovo Institute for Science and Technology, Ulitsa Nobelya 3, Moscow 121205, Russia; tim.ivanov.92@gmail.com; 2 Faculty of Bioengineering and Bioinformatics, Moscow State University 1-73, Moscow 119899, Russia; 3 Faculty of Computer Science, Higher School of Economics, Kochnovskiy Proyezd 3, Moscow 125319, Russia

**Keywords:** mutually exclusive exons, alternative splicing, RNA structure, long-range, duplication, competing structures, evolution, bidirectional control, *Dscam*, *srp*

## Abstract

Alternative splicing is a commonly-used mechanism of diversifying gene products. Mutually exclusive exons (MXE) represent a particular type of alternative splicing, in which one and only one exon from an array is included in the mature RNA. A number of genes with MXE do so by using a mechanism that depends on RNA structure. Transcripts of these genes contain multiple sites called selector sequences that are all complementary to a regulatory element called the docking site; only one of the competing base pairings can form at a time, which exposes one exon from the cluster to the spliceosome. MXE tend to have similar lengths and sequence content and are believed to originate through tandem genomic duplications. Here, we report that pre-mRNAs of this class of exons have an increased capacity to fold into competing secondary structures. We propose an evolutionary mechanism for the generation of such structures via duplications that affect not only exons, but also their adjacent introns with stem-loop structures. If one of the two arms of a stem-loop is duplicated, it will generate two selector sequences that compete for the same docking site, a pattern that is associated with MXE splicing. A similar partial duplication of two independent stem-loops produces a pattern that is consistent with the so-called bidirectional pairing model. These models explain why tandem exon duplications frequently result in mutually exclusive splicing.

## 1. Introduction

Mutually exclusive exon selection, a pattern in which one and only one of tandemly-arranged exons is included in the pre-mRNA, is intrinsic to many eukaryotic genes [1]. While many examples were described in model organisms [2,3], recent reports based on the analysis of RNA sequencing experiments confirm that large multi-cluster mutually exclusive exons (MXEs) are also frequent in higher vertebrates, nematodes and plants [4,5,6] and that they are associated with mutations involved in human pathogenic states [7].

The fact that MXE clusters often exhibit high sequence similarity suggests that they have evolved through tandem exon duplication [8]. In a recent survey, tandemly-duplicated MXE in eukaryotes were predicted based on the properties of exon length and sequence homology [9]. Case studies, such as phylogenetic analysis of tandem exon arrays in metazoan multidrug resistance-associated protein (MRP) genes, revealed multiple independent exon duplications across different phyla and suggested convergent evolution of splicing patterns [10]. On the other hand, non-homologous MXE also exist, suggesting that not all mutually exclusive splicing patterns originate from exon duplications [1].

A number of molecular mechanisms were proposed to explain mutually exclusive selection of exons [1]. If the intron separating two MXE is too short, then the two exons cannot be spliced together because of spatial constraints [6]. This mechanism, however, applies to clusters of only two MXE. Another scenario, which involves both the major and the minor spliceosome, explains mutually exclusive exon choice by the spliceosome incompatibility, but it is limited to quite uncommon clusters of exactly two MXE that have a mixed U2/U12 type of splicing [6]. Next, nonsense-mediated mRNA decay may contribute to mutually exclusive splicing by degrading transcript isoforms with two or more MXE, which induces premature stop codons [11]. This scenario is applicable neither to MXE of multiples of three nucleotides, nor to clusters of more than three MXE, since in the latter case, the lengths can always be combined into a multiple of three nucleotides.

The major mechanism of mutually exclusive splicing is based on competing RNA secondary structures [1]. Comparative sequence analysis of the *Dscam* gene in the *Drosophila* species revealed that the exon 6 cluster, which consists of 48 variable exons, contains a conserved regulatory element (docking site) that is complementary to a set of other conserved regulatory elements (selector sequences) [12]. One and only one of the selector sequences can base pair to the docking site at a time; when the selector sequence upstream of an exon interacts with the docking site, it exposes the intervening exons in a loop, which promotes splicing. A similar splicing pattern associated with competing complementary sequences was found later in exon 4, exon 9 and exon 17 clusters of the same gene [3,13,14,15] and also in other insect genes, including *14-3-3ζ*, *Mhc* and *MRP1* [10,15]. In contrast to the exon 6 cluster of *Dscam*, the docking sites in these other genes are located in the introns downstream of the MXE cluster.

A key component of the model proposed by Graveley (Figure 7 in [12]) is the RNA structure that exposes a group of exons in a loop. While the base pairing between the docking site and the selector sequences excludes the upstream MXE variants and promotes their skipping, some unknown mechanism also activates skipping of the downstream MXE variants, likely through a splicing repressor that prevents them from being spliced together [12]. In a recent work, Yue et al. proposed a mechanism for MXE splicing in the *srp* gene, which is based on two sets of complementary sequences, each surrounding the respective exon [16]. Although these sequences are not competing for a common site, they are important for mutually exclusive exon choice, suggesting that only one of them can form at a time. Similar bidirectional structures have been experimentally confirmed in minigene constructs for the *Dscam* exon 4 cluster in *Apis mellifera* and the exon 9 cluster in silkworm [16]. MXE splicing in hymenopteran *MRP* is guided by an ensemble of multiple intronic stem structures [10]. These examples show that a complex organization of structural regulatory elements could be involved in MXE splicing [16].

The frequent occurrence of competing complementary sequences in genes with MXE suggests that the RNA structure could be a generic mechanism that controls mutually exclusive splicing. Since mutually exclusive splicing is associated with competing RNA structures and also with tandem exon duplication, it raises an intriguing question of whether competing base pairings and tandem exon duplication are related to each other. Here, we propose an evolutionary mechanism, in which a genomic duplication affects an exon along with the adjacent intronic part containing a stem-loop structure. If one of the two arms of the stem-loop is duplicated, it will create two motifs competing for base pairing with the other arm, and the arrangement of competing RNA structures will be exactly identical to that in MXE. Similarly, a partial duplication of two independent stem-loops explains the formation of multiple competing RNA structures in accordance with the bidirectional pairing control model.

## 2. Materials and Methods

### 2.1. Genomes and Genome Annotations

The *Drosophila melanogaster* April 2006 genome assembly was downloaded from the Berkeley Drosophila Genome Project (BDGP, R5) [17]. The respective transcript annotation BDGP5.25.54 was obtained from Flybase [18]. The February 2009 assembly of the human genome (hg19, GRCh37) was downloaded from Genome Reference Consortium [19]. The respective transcript annotation v19 was downloaded from GENCODE [20]. Genome annotations were parsed by a custom script to extract the coordinates of introns and exons. The nucleotide sequences of introns and exons were extracted using the bedtools getfasta tool [21].

### 2.2. Splicing Graph and Mutually Exclusive Exon Datasets

A splicing graph, a bipartite graph, the nodes of which are splice sites and the edges of which are exons or introns of annotated transcripts, was constructed from the respective transcript annotations by using custom scripts. In the splicing graph, we chose pairs of nodes with multiple vertex-independent paths that contain exactly one exon and two introns. Such pairs of vertices correspond to clusters of MXE, and the number of vertex-independent paths between them is equal to the number of MXE in the cluster.

### 2.3. Conservation and Similarity Analysis

As a measure of evolutionary conservation of a genomic interval, we used the proportion of nucleotides in the interval that were identified as evolutionarily conserved by the phylogenetic hidden Markov model (phylo-HMM) [22]. The pre-computed phastConsElements tracks were downloaded from the University of California San Francisco (UCSC) genome browser website (multiple sequence alignments of 100 vertebrates and 15 insects, respectively) [23].

In order to compute the similarity between two sequences, we used local Smith–Waterman alignments implemented in the pairwise2 library in Biopython [24]. We used the pairwise2.align.localms function with the following scores: match=1, mismatch=-0.2, gapopening=-1 and gapextension=-0.5. Sequences longer than 10,000 nucleotides were not considered. The normalized similarity measure σ between two sequences, *x* and *y*, was computed as:

(1) $$\sigma \left(x,y\right)=\frac{s(x,y)}{\max\{s(x,x),\;s(y,y)\}},$$

where s(x,y) is the score of the local alignment of *x* and *y*. This measure takes values between 0 and 1 and attains its maximum value when the two sequences are identical.

### 2.4. Choice of Control Sequences

For each nucleotide sequence, we generated a shuffled control sequence that has exactly the same dinucleotide frequencies as the given sequence, by using the following simple routine [25]. Given three consecutive nucleotides x,y,z, we searched for an occurrence of the dinucleotide x,z and exchanged them so that x,y,z becomes x,z and vice versa. By construction, the dinucleotide frequencies are not affected by this procedure. This permutation is repeated *l* times, where *l* is the length of the sequence.

Another control procedure was to replace the given intron (exon) by a randomly-chosen intron (respectively, exon) matched by the length. To implement this, the nucleotide sequences in each class (intron, exon) were sorted by ascending length, and then, the rank of the sequence was altered randomly by an offset not exceeding ±10 positions.

### 2.5. RNA Structure Analysis and Free Energy Calculation

The RNA structure associated with MXE belongs to the long-range class of structures, and therefore, it has to be considered intermolecular [26,27]. The following programs from the ViennaRNA package [28] were used: RNAup, which models the thermodynamics of RNA-RNA interactions as the additive contribution of the energy necessary to open the binding site and the energy gained from the hybridization [29], and RNAplex, a tool that allows one to quickly find possible hybridization sites for two RNAs, but uses precomputed accessibility profiles with an approximate energy model [30]. Both programs were run with the –noLP key to suppress lonely base pairs. The programs were run on pairs of true sequences and then run again on pairs of control sequences, either shuffled by preserving dinucleotide content or chosen randomly from the same class (intron, exon) when matched by the length. The difference of MFEs, $\Delta \Delta G=\Delta G-\Delta G_{0}$, where ΔG is the MFE for the true pair and $\Delta G_{0}$ is the MFE for the control pair, was used as the measure of the propensity of two nucleotide sequences to base-pair. The values of ΔΔG larger than 30 kcal/mol by the absolute value were discarded. In order to focus the analysis of MFE on the conserved part of the sequence, we intersected the sequence of interest with the phastConsElements track using the intersectBed program and took the concatenation of its conserved parts. Complementary regions were visualized using the UCSC Genome Browser [23].

### 2.6. Statistical Analysis

The data were analyzed and visualized using R statistics software Version 3.4.1 and the ggplot2 package. Statistical tests were performed without the finite population size correction factor on matched samples of genomic intervals related to MXE, viewed as simple random samples taken from the universe of all such genomic intervals related to other exon classes. One-sided *p*-values are reported throughout the paper.

## 3. Results

**Figure 1:**
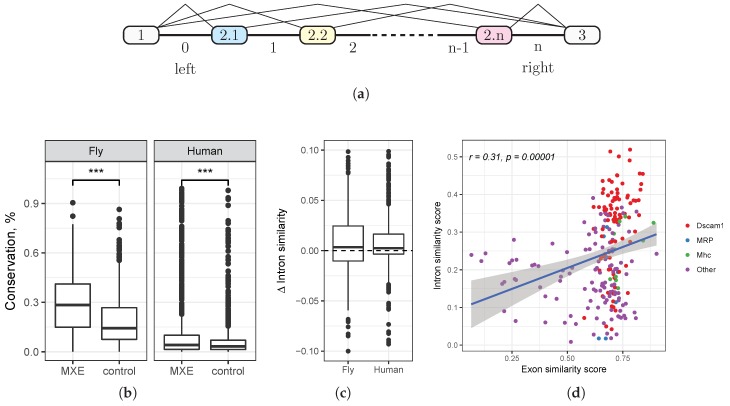
(**a**) A schematic representation of the mutually exclusive exon (MXE) cluster. Exons 1 and 3 are constitutive; exons 2.1,2.2,⋯,2.n are mutually exclusive. The intervening introns are numbered from 0–*n*. The terminal flanking introns are called “left” and “right”, respectively. (**b**) The conservation rate in introns flanking MXE and in a random sample of introns flanking other exons (see the Methods). (**c**) The difference of similarity score $\Delta \sigma =\sigma -\sigma_{0},$ where σ is the similarity score of consecutive introns *i* and i+1 flanking MXE, and $\sigma_{0}$ is the respective similarity score in the control sample of introns matched by the length. (**d**) The similarity score of consecutive introns *i* and i+1 vs. the similarity score of consecutive exons *i* and i+1.

Throughout this paper, we use the notation illustrated in Figure 1a. Each cluster of MXE is located between two flanking constitutive exons (exons 1 and 3) and consists of *n* alternative exons, denoted 2.1,⋯,2.n, and n+1 intervening introns, which are numbered from 0–*n*. The introns flanking the constitutive exons, i.e., the introns 0 and *n*, are also denoted as “left” and “right” (Figure 1a). The coordinate system is strand-independent, i.e., the left intron is closer to the 5’-end of the pre-mRNA than is the right intron.

### 3.1. The Sample of Mutually Exclusive Exon Clusters

Large annotated sets of MXE clusters are available through databases of transcript annotations in human, fruit fly and worm. Among these organisms, only fruit fly and human have a sufficient number of MXE for statistical analysis. To avoid redundancy, the mammalian and insect orthologs of human and fruit fly genes with MXE were not included in this study because their MXE annotations rely on cross-species transcript comparisons.

The majority of annotated MXE clusters consist of only two exons (see the procedure in the Section 2.2). In *D. melanogaster*, there are 126 MXE clusters; of these, 102 consist of two exons, and the following genes have clusters of three or more MXE (the number of exons is in parenthesis, in descending order): *Dscam* (48,33,12), *MRP* (8), *Mhc* (5,3), *TepII* (5), *Atp-α* (4), *slo* (3), *wupA* (4), *14-3-3ζ* (3), *babo* (3), *Pfk* (3) and *Esyt2* (3). In human, the transcript annotation contains 526 two-exon MXE clusters; all other annotated clusters contain three MXE: *CACNA1E*, *CACNB2*, *CADPS2*, *CNOT10*, *GMDS-AS1*, *HMGN2P28*, *hsa-mir-7515*, *LIX1L*, *LY6G5C*, *NBPF14*, *PIGFP1*, *RFT1*, *RP11-132M7.3*, *RP11-315I20.1*, *RP11-499P20.2*, *RP11-894J14.5*, *RP5-1101C3.1*, *SMARCA4*, *TBX18* and *ZFP2*. In *D. melanogaster*, competing RNA structures associated with MXE clusters have been reported for *Dscam* [3,12,13,15,31], *MRP* [10], *Mhc* [15], *srp* [16] and *14-3-3ζ* [15]. It is our hypothesis that MXE in other genes tend to be regulated by competing RNA secondary structures.

### 3.2. Evolutionary Conservation in Introns Flanking Mutually Exclusive Exon

We first asked whether the degree of evolutionary conservation is different in introns flanking MXE compared to introns flanking other exon classes. Instead of directly using phastCons, a metric derived from phylogenetic hidden Markov model (phylo-HMM) analysis of whole-genome alignments [22], we computed the fraction of nucleotides in the intron that belong to phastConsElements, which is a good proxy for the average conservation rate across genomic ranges [23]. We computed this fraction for each intron flanking MXE and compared it to the respective fraction for a randomly-chosen intron of the same gene (Figure 1b).

On average, 29.0% (respectively, 10.5%) nucleotides are evolutionarily conserved in introns flanking MXE in *D. melanogaster* (respectively, human), while the corresponding figure for a randomly-chosen intron is 18.3% (respectively, 6%). In both cases, the difference is highly statistically significant (Mann–Whitney test, $p-\mathrm{value}<10^{-6}$). That is, we observe an increased amount of selection in introns flanking MXE, suggesting that they may harbor functional regulatory elements that mediate mutually exclusive splicing.

### 3.3. Similarity of Introns Flanking Mutually Exclusive Exon within a Cluster

It is believed currently that some MXE clusters have appeared evolutionarily through tandem genomic duplications [8]. Indeed, exons within MXE clusters are often homologous and have similar lengths [7]. While exon similarity is maintained due to protein-coding constraints, a much weaker selection is acting on intronic sequences. Here, we ask whether traces of similarity between consecutive introns also remained in MXE clusters.

In order to capture short similar motifs, we computed the similarity score $\sigma (x_{i},x_{i+1})$ for each pair of consecutive introns flanking MXE using local sequence alignment and normalized it to the maximum value that it could have obtained if the two sequences were exactly identical (see Section 2.3). The longer is the common motif in the two sequences, the larger is the similarity score. As a reference, we computed the respective figures of the similarity score for a control sample of introns flanking exons that are not MXE. We expected that short similar motifs, if they exist, will systematically bias the similarity score of MXE introns towards larger values compared to that of other introns.

Indeed, the sample of consecutive introns that flank MXE had a significantly larger median similarity score than did the control sample (Mann–Whitney test, $p-\mathrm{value}<10^{-6}$). Since longer introns are more likely to contain similar motifs just by chance, we chose a control sample that was matched by intron length and analyzed differences in similarity scores (Figure 1c). The median difference of similarity scores was significantly greater than zero (Wilcoxon test, $p-\mathrm{value}<10^{-5}$ in fruit fly, $p-\mathrm{value}<10^{-10}$ in human), however small by the absolute value (the average difference of about 2% and 1% in fruit fly and human, respectively). The positive shift of the similarity score indicates that consecutive MXE introns contain more homologous regulatory elements such as selector sequences than do other introns. The small absolute value of this shift is not unexpected, as it is on the order of magnitude of 1–2% for a conserved 14-nt stem structure in a 600-nt intron [32].

We also found that the degree of similarity of two consecutive MXE introns positively correlates with the degree of similarity of their adjacent exons (r=0.31, Figure 1d). In fruit fly, this correlation remains significant (r=0.18, n=139, *t*-test, p-value=0.017) even after removing the cluster of highly similar consecutive exons of *Dscam*. That is, the more similar are the consecutive MXE, the more similar are their flanking introns. Since some MXE are generated through tandem duplication, this observation indicates that the duplication also affected a part of the adjacent intron.

In sum, we observed that pairs of consecutive introns flanking MXE are, on average, more similar to each other than are pairs of introns flanking other exon classes, and the degree of this similarity correlates with the similarity of adjacent exons. These observations suggest a possibility that a part of the adjacent intron space has been duplicated together with the exon and remained under selective constraint, which as we have shown in Section 3.2, is high in introns flanking MXE.

### 3.4. Complementary Pairings between Introns Flanking Mutually Exclusive Exon

**Figure 2:**
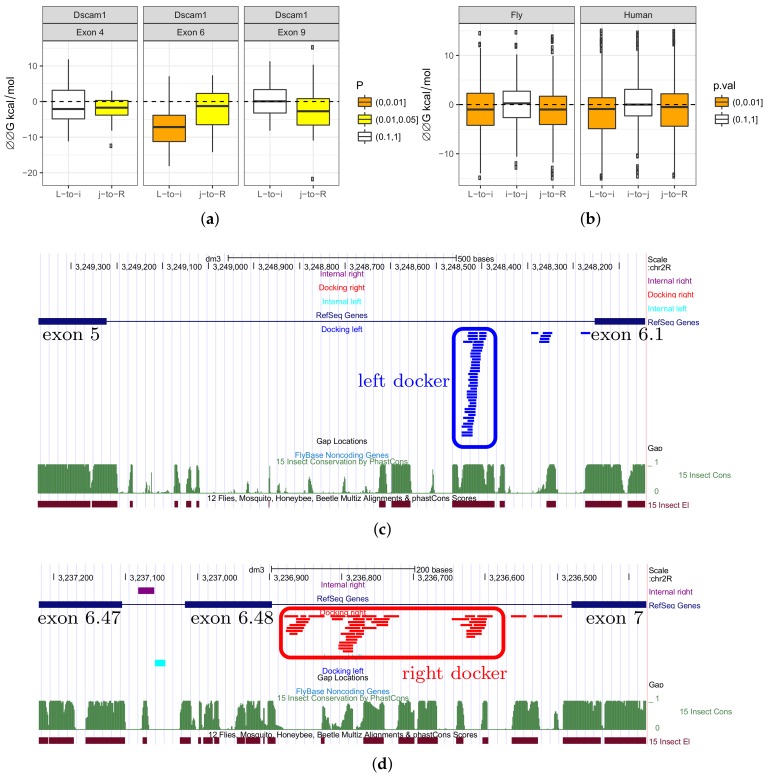
(**a**) The distribution of the minimum free energy (MFE) differences $\Delta \Delta G=\Delta G-\Delta G_{0},$ where ΔG is the MFE of hybridization of the actual pair of introns (L-to-i and j-to-R), and $\Delta G_{0}$ is the respective figure for shuffled sequences, for the three largest MXE clusters of *Dscam*. (**b**) The distribution of the minimum free energy (MFE) differences for the actual pair of introns (L-to-i, i-to-j, and j-to-R) in fruit fly and human MXE clusters with two exons. Color codes show the significance according to the Wilcoxon signed rank test. (**c**,**d**) The predicted docker sequences in the left intron (blue) and in the right intron (red).

Since introns flanking MXE are under stronger selection than other introns, it is reasonable to ask whether it could be due to complementary base pairings. We first applied the RNAup program to all pairwise combinations of the left and right introns vs. internal introns in three large MXE clusters (exon 4, exon 6 and exon 9) in *D. melanogaster Dscam*. We predicted the minimum free energy (MFE) of hybridization of each pair and then applied the same procedure again to dinucleotide-shuffled sequences. The difference between MFEs of the original and of the shuffled pair, ΔΔG, reflects the propensity of the two sequences to base pair (Figure 2a). Negative values of ΔΔG indicate that the actual sequences fold into more stable structures than do shuffled sequences.

Consistent with what is known about competing RNA structures in *Dscam*, we observed a tendency for it to form an RNA structure in all three MXE clusters. While the exon 4 cluster (12 exons) and exon 9 cluster (33 exons) seemed to contain the docker site in the right intron in agreement with the previous findings [15], exon 6 cluster (48 exons) had a significant propensity to base pair internal introns by docker sites in both left and right introns.

We next looked into specific RNAup predictions for intramolecular base pairings within the exon 6 cluster of *Dscam* (Figure 2c,d). In the left intron, RNAup correctly identified the unique docker sequence that was reported previously, while complementary base pairings of the internal introns with the right intron resulted in several docker candidates. Notably, the right docker sequences were not scattered through the intron, but formed several clusters that also overlap conserved regions. This observation is concordant with the bidirectional control model that was proposed for the exon 4 and exon 9 clusters of *Dscam*, albeit in different species [16].

Next, we asked if the left and the right introns had a tendency to base-pair internal introns in other MXE clusters. However, RNAup is not very effective for long sequences. Another program, RNAplex, which does not account for the intramolecular structure, can be used to detect sequence complementarity in the same setting of comparing MFE of hybridization of two introns to that of two shuffled control sequences (Figure 2b). We applied RNAplex to conserved parts of intronic sequences and observed a statistically-significant change when predicting the MFE of hybridization for the left and right introns with internal introns (Wilcoxon test, p-value<0.005), but notably not for base-pairing of internal introns with each other (p-value≃0.45).

In sum, we found that in annotated MXE clusters, there was a tendency for the left and the right introns to base-pair internal introns, but not for the internal introns to base-pair each other. This suggests that the mechanism of mutually exclusive exon selection that is based on docker and selector sequences, which was proposed for mutually exclusive splicing in *Dscam*, could be common in many other MXE clusters. This, and traces of similarity between consecutive introns suggest that, perhaps, there is a mechanism responsible for the convergent evolution of MXE splicing that is related to their evolutionary origin through tandem genomic duplications.

## 4. Discussion

From the evolutionary perspective, exon duplication can be regarded as a way of generating functional diversity of proteins, along with gene duplication [8]. It can occur through a variety of mechanisms, including transposon-mediated events and non-homologous recombination [33]. The outcome is that a part of the gene sequence is tandemly duplicated, along with regulatory signals that determine exon-intron structure.

In this work, we demonstrated that introns flanking MXE tend to be more conserved than do other introns, that they are more similar to each other within the MXE cluster than are random intron sets matched by the length and that the degree of this similarity grows with increasing similarity of the adjacent exons. This indicates that the genomic duplication affected both exonic and intronic regions. Moreover, we showed that the first and the last introns in the cluster have a high propensity to hybridize internal introns. Taken together, these pieces of evidence suggest the following model.

**Figure 3:**
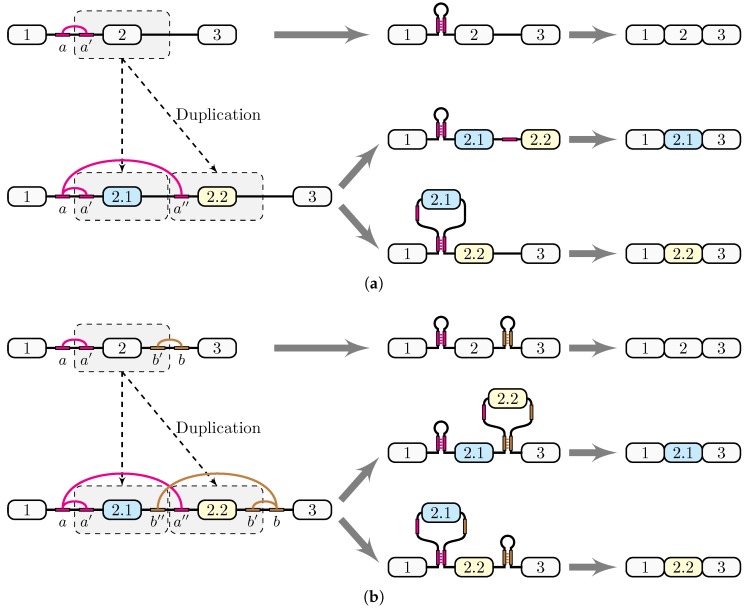
Generation of competing RNA structures through a genomic duplication. (**a**) If a genomic duplication affects an exon and one arm of the stem-loop located in the flanking intron, it creates a pair of competing complementary sequences that loop out exon 2.1. (**b**) If the genomic duplication affects the exon and the adjacent parts of its two flanking introns, each containing one arm of a stem-loop, it will create a pair of competing complementary sequences, each looping out one of the exons 2.1 and 2.2. Notably, the arrangement of complementary parts is exactly as suggested in the bidirectional model in the *srp* gene [16].

Consider a genomic duplication affecting the genomic region that contains an exon (exon 2) and an intron (Figure 3a). Assume additionally that the intron spanning between exons 1 and 2 contains a pair of complementary sequences, *a* and $a^{\prime }$, which are capable of forming a stem-loop structure, and that only one of the two complementary parts, $a^{\prime }$, is affected by the duplication. Stem structures often appear in eukaryotic introns as structural units related to constitutive and alternative splicing [34]; they can, in fact, span large distances and function as elements approximating distant splice sites [35].

As a result of the duplication, the two exon 2 copies are arranged tandemly, while $a^{\prime }$ and its copy $a^{\prime \prime }$ are both complementary to *a* (Figure 3a). This creates a pair of competing RNA structures, in which *a* is paired either to $a^{\prime }$ or to $a^{\prime \prime }$; in the former case, exon 2.1 is included, while in the latter case, it is placed in a loop and skipped. This scenario gives a plausible explanation of how docker and selector sequences can emerge through genomic duplications.

The molecular mechanism of MXE inclusion in the docker-selector model, however, is more sophisticated than the formation of competing structures in the pre-mRNA. It is regulated by the interplay between splicing activators and repressors, their respective *cis*-regulatory elements and splice site strengths [36]. A combination of competing RNA structures, a globally-acting splicing repressor, *hrp36*, and weaker splice sites of MXE cooperatively keep most of the alternative exon variants inactivated. The docker-selector interaction activates the target exon by promoting the recognition of its splice sites, likely as a result of spatial approximation or repression release. This model, however, provides a mechanistic explanation only for splicing of one of the two alternative introns. It is believed that the second splicing choice, i.e., one not related to docker-selector interaction, could be determined by splice site strengths [36].

Recently, Yue et al. proposed the so-called bidirectional RNA pairing model based on the RNA structure within the exon 4 cluster of *srp* [16]. In this gene, two pairs of conserved complementary intronic elements surround two alternative exons. Each of the two pairs contributes to the activation of the respective target exon. The base pairings of the two structures are not mutually exclusive, but their simultaneous formation is impeded by a pseudoknot. A similar bidirectional model applies to other genes such as *Drosophila RIC-3* and *MRP1*, the exon 4 cluster of hymenopteran and exon 9 cluster of lepidopteran *Dscams* [10,16]. Therefore, the regulation based on multiple docker-selector structures may represent a generic mechanism for the regulation of mutually exclusive splicing.

A slight modification of the scenario shown in Figure 3a provides an evolutionary mechanism that could generate bidirectional competing RNA structures. Consider a genomic duplication affecting an exon and also its two flanking introns, which contain two pairs of complementary sequences, *a* and $a^{\prime }$, and $b^{\prime }$ and *b* (Figure 3b). As a result of this duplication, two exon 2 copies will be again arranged tandemly, and two competing RNA structures, $a-a^{\prime }$ vs. $a-a^{\prime \prime }$, and $b^{\prime }-b$ vs. $b^{\prime \prime }-b$, will be created. Notably, they will be arranged so that $b^{\prime \prime }$ is located upstream of $a^{\prime \prime }$, as is the case in the *srp* gene [16]. Although each pair of competing structures can form independently of the other pair, not all four combinations are equally likely because of the pseudoknot (Figure 3b). If *a* pairs $a^{\prime }$ and $b^{\prime \prime }$ pairs *b*, then exon 2.2 is placed in a loop and skipped. Conversely, if *a* pairs $a^{\prime \prime }$ and $b^{\prime }$ pairs *b*, then exon 2.1 is looped out and skipped. It is still possible that *a* pairs $a^{\prime }$ and $b^{\prime }$ pairs *b* so that neither exon is looped out, leading to a simultaneous inclusion of both exons. Unlike unidirectional model (Figure 3a), the bidirectional competing model explains mechanistically the suppression of MXE variants both upstream and downstream of the target exon.

The docker-selector model of mutually exclusive splicing admits two variants. In the first variant, the docker site is located upstream of the MXE cluster in the left intron, as it is in the exon 6 cluster of *Dscam*. In the other variant, the docker site is located downstream of all MXE in the right intron, as it is in *14-3-3ζ*, *Mhc*, *MRP1* and other MXE clusters [10,15]. The bidirectional competing model admits both of these variants at the same time. The increase in the conservation level and in the propensity to form competing RNA structures is not the same for the left and for the right introns, with slightly higher figures for the right intron (data not shown). A question naturally appears at this point: why do some genes prefer left docker sites, while others use right docker sites to regulate MXE splicing?

While there is no obvious answer to this question, a fundamental difference between left and right dockers from the biochemical point of view could be in the regulability of their pairings with the selector sequences. Since the RNA structure forms co-transcriptionally [37], there must be a kinetic advantage for $a^{\prime }$ to base pair *a*, as compared to $a^{\prime \prime }$ if the docker site is located in the left intron, as compared to the case when $b^{\prime \prime }$ and $b^{\prime }$ are transcribed sequentially and obtain equal chances of base pairing with *b*, which appears last if the docker site is located in the right intron. Although it could explain the prevalence of the right docker sites in the genes, in which docker-selector systems have been identified, many other factors influence the kinetics of co-transcriptional folding and splicing of the pre-mRNA [38].

Another important factor that could influence the formation of mutually exclusive conformations is the thermodynamics of the RNA structure. While $a^{\prime }$ and its copy $a^{\prime \prime }$ (Figure 3) can form equally-stable duplexes with *a*, other structural elements such as loops, b-b’ helices and pseudoknots also contribute to the MFE of the pre-mRNA. It is debatable whether the MFE of the RNA structure could influence the rate of exon inclusion. On one hand, the ratio of alternative isoforms in *Gug* pre-mRNA changes with increasing MFE of the duplex [32]. On the other hand, May et al. related the MFE of the RNA structure with the log-fold change of exon inclusion in *Dscam* minigenes and found that the correlation is not significant [3]. The relationship between MFE and exon inclusion in vivo must be even more complex because docker-selector systems have evolved since the time of the duplication to adapt novel biological functions. The analysis of naturally-occurring switches between mutually exclusive pre-mRNA conformations is the matter of further investigations.

## 5. Conclusions

Annotated MXE clusters have distinct features such as an increased level of selection and similarity and a high propensity to form competing RNA structures. We propose an evolutionary mechanism for the generation of such competing structures via genomic duplications that affect exons and their adjacent introns. A duplication copies one of the two arms of an intronic stem-loop, thus generating two selector sequences that compete for the same docking site, a pattern that is assumed to drive MXE splicing. Similarly, a partial duplication of two independent stem-loops can produce a pair of competing RNA structures, consistent with the bidirectional pairing control model. This could be a generic mechanism that explains why tandem exon duplications frequently result in a mutually exclusive splicing pattern. It suggests that the MXE splicing that is based on docker and selector sequences, as in the *Dscam* gene, could be more widespread than is believed currently.
